# Lersivirine: a new NNRTI active across HIV-1 subtypes with a unique resistance profile

**DOI:** 10.1186/1758-2652-13-S4-O49

**Published:** 2010-11-08

**Authors:** J Mori, M Westby, M Tawadrous, E van der Ryst, C Charles

**Affiliations:** 1 Pfizer Global Research and Development, Sandwich Laboratories, Sandwich, UK; 2 Pfizer Global Research and Development, New London, USA

## Purpose of the study

Lersivirine (UK-453,051), a new potent NNRTI, displays novel binding with a unique *in vitro* resistance profile, and shows potential for use against transmitted NNRTI resistant virus and as a candidate for sequential NNRTI therapy. Further *in vitro* analyses have been performed to establish the breadth of activity and identify potential populations that might benefit from lersivirine therapy. Lersivirine activity against various HIV-1 subtypes and viruses with decreased susceptibility to etravirine (ETR) were characterised.

## Methods

The PhenoSense™ assay was used to assess the antiviral activity of lersivirine against different HIV-1 subtypes (A, A1, B, BF, C, C/H, D, F, F1, G and H), and the circulating recombinant forms (CRFs) CRF01_AE and CRF02_AG. All were obtained from treatment-naïve patients. Nineteen additional clinical viruses with NNRTI resistance associated mutations (RAMs) were selected based on their reduced susceptibility to ETR.

## Summary of results

Lersivirine was active against a panel of 80 clinically- derived viruses representing subtypes A to H, including several CRFs, from a range of geographical origins (geometric mean IC_50_ fold change [FC] to reference virus was 0.92). IC_50_ FC were < 2 for all viruses with the exception of 1 subtype BF and 1 subtype C (geometric mean IC_50_ FC: subtype BF = 0.98, 95% CI 0.64 - 1.49, n=7; subtype C = 1.07, 95% CI 0.88 - 1.30, n=25). Lersivirine retained activity (< 10 FC IC_50_) for 11 of the 19 viruses with ETR resistance (> 2.9 FC IC_50_, lower clinical cut-off). Overall, a direct correlation between lersivirine and ETR susceptibility was not found (R^2^ = 0.002). This is consistent with different genotypic resistance profiles. Indeed to date, reduced susceptibility to lersivirine and ETR is associated with the presence of different specific NNRTI RAMs.

## Conclusions

Lersivirine showed comparable activity across a range of viruses representing subtypes A to H. The activity of lersivirine against ETR- resistant viruses reflects significant differences in the resistance profiles of lersivirine and ETR consistent with the unique binding of lersivirine in the NNRTI binding pocket. Lersivirine has a distinctive *in vitro* resistance profile and may provide an additional therapy choice for patients with evidence of NNRTI resistance.

